# Association of *ABCG2* Polymorphisms with Methotrexate Efficacy and Toxicity in Saudi Rheumatoid Arthritis Patients

**DOI:** 10.3390/ph18091365

**Published:** 2025-09-12

**Authors:** Zeina W. Sharawi, Lina A. Alqurashi, Ahlam M. Alharthi, Ibtisam M. Jali, Maha H. Jamal, Yasser Bawazir, Mohammad Mustafa, Sami M. Bahlas, Hassan Daghasi, Talal S. Alharthi, Dalal Sameer Al Shaer, Rania Magadmi

**Affiliations:** 1 Department of Biological Sciences, Faculty of Science, King Abdulaziz University, Jeddah 21589, Saudi Arabia; 2 Department of Clinical Pharmacology, Faculty of Medicine, King Abdulaziz University, Jeddah 21589, Saudi Arabia; 3 Department of Medicine, Faculty of Medicine, King Abdulaziz University, Jeddah 21589, Saudi Arabia; 4 Rheumatology Unite, Department of Medicine, Faculty of Medicine, University of Jeddah, Jeddah 21589, Saudi Arabia; 5 Department of Rheumatology, Internal Medicine Department, Al Hada Armed Forces Hospital, Taif 26792, Saudi Arabia; 6 Department of Pharmacy, King Abdulaziz Specialist Hospital, Ministry of Health, Taif 26792, Saudi Arabia; 7 Department of Genetic Medicine, Faculty of Medicine, King Abdulaziz University, Jeddah 21589, Saudi Arabia

**Keywords:** rheumatoid arthritis, methotrexate, *ABCG2*, pharmacogenetics, single-nucleotide polymorphism

## Abstract

**Background/Objectives**: Methotrexate (MTX) is currently the most widely used treatment for Rheumatoid Arthritis (RA) due to its demonstrated efficacy and well-known safety profile. However, the effectiveness and toxicity of MTX can vary among patients, partly due to genetic factors. Therefore, this study aimed to investigate the associations between the polymorphisms in the ABC subfamily G member 2 (*ABCG2*) gene and MTX effectiveness/toxicity in Saudi Arabia RA patients. **Methods**: The study is a retrospective, multicenter, case–control study that uses Sanger sequencing techniques for genotyping. **Results**: More than half of the patients (55.56%) were poor responders, with a slightly higher mean age. However, there was no significant difference between the two groups, not only in terms of age but also in other demographics and clinical factors. Regarding the rs2231137 polymorphism, the CC, CT, and TT genotype frequency were 91%, 7%, and 2%, respectively. The mutated variant (TT) was only observed in the positive rheumatoid factor group. Notably, none of these genotypes displayed any significant correlation with demographic characteristics, clinical features, or MTX efficacy/toxicity. **Conclusions**: This study is the first pharmacogenetic study of rs2231137 polymorphism in RA patients utilizing linear regression, revealing that rs2231137 polymorphism is not a predictor of either MTX efficacy or toxicity in RA patients. Therefore, more research is needed.

## 1. Introduction

Rheumatoid arthritis (RA) is a chronic systemic autoimmune disorder that causes symmetrical polyarthritis, with a global prevalence of up to 1.7% among adults, women in particular. The disease is often progressive; it begins in small joints and progresses to involve major ones, causing pain, stiffness, swelling, and functional loss that leads to disability and early death [1,2].

There is no cure for RA, so early use of disease-modifying anti-rheumatic drugs (DMARDs) represents the core component of RA management. In clinical practice, methotrexate (MTX) represents the first and best choice for RA treatment despite the availability of several conventional DMARD options [3,4,5], because of its ability to reduce disease development in the short term and, in some RA patients, delay or stabilize the progression of bone erosion over the longer term [6,7]. Within the Arab region, reports indicate that MTX is the most commonly used DMARD among RA patients, with a satisfactory therapeutic maintenance level [8,9]. However, MTX treatment still causes significant response diversity in patients, and 32% of patients experience adverse drug reactions (ADRs), which may lead to interrupting their treatment with MTX [10]. The variety in responses may be caused by several factors, including higher body mass index, smoking, and gene variation, particularly single-nucleotide polymorphisms (SNPs) in genes encoding membrane transporter proteins for MTX, such as ATP-binding cassette (ABC), which function as MTX efflux [11,12].

ABC subfamily G member 2 (*ABCG2*) is an ABC transporter protein and efflux pump for MTX. ABCG2, known as breast cancer resistance protein (BCRP), is located in chromosome 4q22, encoded by the *ABCG2* gene [13]. It is normally localized in the small intestine, in bile canalicular membranes of hepatocytes, and in renal proximal tubes. Also. export MTX into the urine, bile, or back into the intestinal lumen [11]. Genetic variations in this gene may have the potential to impact the transport efficiency of ABCG2, leading to variations in pharmacokinetics or drug resistance [14,15]. Research aimed at studying the role of the *ABCG2* SNPs found that the *BCRP 34G>A* SNP (rs2231137) decreased BCRP transporter activity due to a disruption in the localization of the apical plasma membrane [15]. Also, it found that individuals carrying the *BCRP* 421C>A SNP (rs2231142) exhibit reduced protein levels compared to those with the wild-type variant (421CC). Consequently, normal cells became more sensitive to chemotherapeutic agents (e.g., MTX) [16]. Additionally, in vivo investigations indicate that the transport activity of the intestinal BCRP in 421AA individuals is approximately 23% of that observed in 421CC individuals [17]. rs2231137 and rs2231142 are missense genetic variants in the ABCG2 gene, where the G nucleotide is substituted with an A at position 34, and a C nucleotide is substituted with an A at position 421, respectively, with a high allelic frequency in Asian populations according to the National Center for Biotechnology Information (NCBI).

Despite numerous studies in pharmacogenomics, few have investigated the relationship between *ABCG2* and MTX toxicity in patients with RA, specifically the rs2231137 SNP. Moreover, research on the relationship between *ABCG2* and MTX response in RA patients is limited in Saudi Arabia. This gap reflects the need for targeted research to optimize therapeutic strategies for this population. Therefore, this study aimed to investigate the associations between *ABCG2* SNPs and the efficacy and toxicity of MTX in Saudi Arabian RA patients. Discovering how genetic variation affects RA patients’ response to MTX would enhance disease control and reduce the risk of ADRs, leading to improved quality of life.

## 2. Results

### 2.1. Clinical and Demographic Data of Participants

Table 1 display the baseline characteristics of all participants. The study consisted of 99 RA, of which 78 (78.78%) were female and 21 (21.21%) were male. The mean age was 52.7 years, and 6 (6%) participants were non-Saudi residents. Approximately 58.58% of the patients reported using a steroid or non-steroid anti-inflammatory drug (SAIDs/NSAIDs). Notably, the percentage of good responders (44.44%) and poor responders (55.56%) did not differ significantly, and among all ADRs, gastrointestinal (GI) upset (7%) was the most commonly reported.

### 2.2. Demographic Data and Clinical Parameters as Predictors of MTX Response in RA Patients

The demographic data and clinical parameters as predictors of MTX response in RA patients are shown in Table 2. An independent *t*-test was performed to determine whether the mean age differed between good responders and poor responders. As a result, there was no significant difference between the two groups. However, the mean age of poor responders was slightly higher.

Regarding the other factors (Gender, nationality, ADRs, MTX duration, and taking SAIDs/NSAIDs) a chi-square test was performed and found that there was no statistically significant relationship between any of these factors and the two groups.

### 2.3. Genotype Distributions and Allele Frequencies of ABCG2 Polymorphisms

The *ABCG2* polymorphisms, genotype distributions, and allele frequencies are presented in Table 3. The results of rs2231137 (G34A) polymorphism showed that the frequency of the CC genotype was 91%, the heterozygous genotype (CT) frequency was 7% and the homozygous genotype (TT) frequency was 2%. Also, the C allele frequency was 94%, while the T allele was 6%.

The rs2231142 (C421A) SNP was not included in the subsequent data analysis as it was found in only one patient as a heterozygous genotype.

### 2.4. Associations Between ABCG2 SNP (rs2231137) and Patient Response to MTX

Table 4 summarizes the associations between rs2231137 SNP and patient response to MTX. The result shows that the CT genotype appears to almost quintuple the possibility of poor response to MTX. However, the differences were statistically insignificant between good and poor responders. Moreover, there was no significant difference in the allele frequency either.

Next, Table 5 summarizes the analysis of genotype distributions based on different inheritance models to assess the association between carrying C allele or T allele and patients’ response to MTX. However, no significant correlation was found between patient response to MTX and any models.

### 2.5. Associations Between ABCG2 SNP (rs2231137) and Developing ADRs

Among all RA patients included in the genotyping, the most frequent ADR was GI upset (7%). As shown in Table 6, participants with TT genotype did not develop any ADRs compared to other genotypes. However, the results revealed that there are no significant associations between any ADRs and rs2231137 SNP genotypes.

### 2.6. Associations Between ABCG2 SNP (rs2231137) Genotypes with RF

The relationship between G34A SNP genotypes (rs2231137) with Rheumatoid factor (RF) is shown in Table 7. It was found that the mutated variant (TT) was only observed in the positive RF group. However, in the statistical analysis, there was no significant association between rs2231137 SNP genotypes and the positive and negative RF.

### 2.7. Association of ABCG2 SNP (rs2231137) Genotypes with ESR Level and MTX Dose in the Patients

The results show that the erythrocyte sedimentation rate (ESR) level means for genotypes CC, CT, and TT were equal to 27.52, 22, and 33.5, respectively. Also, the mean MTX dose was equal to 15.16, 13.57, and 15 for CC, CT, and TT, respectively, as shown in Table 8. However, there was no statistically significant relationship between the ESR level mean or MTX dose mean with genotypes CC, CT, and TT.

### 2.8. Association of ABCG2 SNP (rs2231137) Genotypes with the Disease Activity

Table 9 displays the DAS-28 means for genotypes. The CC genotype was equal to 3.07, and the CT and TT genotypes were equal to 3.88 and 2.43, respectively. There was no statistically significant association between the genotype group and the Disease Activity Score-28 (DAS-28) mean.

## 3. Discussion

In recent years, the pharmacogenetic studies of DMARDs in treating patients with RA have gained significant attention, particularly MTX, which is a folate anti-metabolite drug that represents the anchor drug for RA treatment. However, despite its demonstrated efficacy and well-known safety profile, MTX treatment still causes significant response diversity among RA patients. According to estimates, up to 50% of RA patients fail to reach their treatment goals, while up to 32% develop ADRs such as stomatitis, gastrointestinal upset, ulcers, liver toxicity, pancytopenia, and pneumonitis, which may lead to interrupting their treatment with MTX [10]. Consequently, in this study, the aim was to determine the relationship between the responses to MTX and the rs2231137 SNP of the *ABCG2* gene. Also, the study will explore the relationship between rs2231137 SNP genotyping data and ESR, ADRs, DAS-28, and RF.

In this study, it was observed that the mean age of the RA patients was 52.7 years; this finding was consistent with the systematic analysis study of the disease global burden conducted in 2021, where RA was found to affect middle-aged and older individuals mostly [18]. Also, the results showed that the majority of the patients were female (78.78%), aligning with the fact that RA has a higher prevalence in females compared to males [18]. Investigations suggest that hormonal factors, particularly estrogen, play a significant role in RA development as a result of their immunomodulatory effects. Moreover, the hormonal changes in women during pregnancy, breastfeeding, menopause, and oral contraceptive use may influence disease activity [19].

The results showed that GI upset is the most common ADR in RA patients, accounting for 7% of cases in comparison to other ADRs. Similarly, it was observed in Al-Malaq et al.’ study, which followed 186 RA patients to document the ADRs of MTX in Saudi RA patients and found that GI upset was the most frequent ADR among the patients [20]. Moreover, the study demonstrated that females were more susceptible to ADRs compared to males, and this result is in agreement with the findings revealed by Mustafa et al. in their study [10]. The similarity in the results across the studies confirms that GI upset is the most common ADR of MTX in RA patients.

Also, the study delved into the effectiveness of MTX, and it was discovered that the percentage of poor responders was higher than good responders, with 55.55% and 44.44%, respectively. Interestingly, such findings differ from previous studies’ reports, which indicate that good responders are more prevalent than poor responders [21,22]. The reason behind the variance with the results of previous studies may lie in the fact that they used different criteria to define a “good responder”. This study classified a patient as a “good responder” only if they achieved full remission in about 3–6 months, whereas other studies included partial remission in their definition of a good responder. Furthermore, there was no significant difference between the two groups based on age, and this result is in agreement with the result of a study that enrolled 775 RA patients [23]. However, herein, the mean age of poor responders was slightly higher.

Regarding the relationship between rs2231137 SNP with MTX efficacy and toxicity in RA patients, the results did not yield any significant relationship between the responses to MTX and the rs2231137 SNP genotypes/allele frequency, where the *p*-value was 0.21, 0.25, respectively. Moreover, there were no significant associations between rs2231137 SNP genotypes and ADRs or the other clinical data (ESR, RF, DAS-28, MTX dose, and MTX duration), suggesting that the rs2231137 SNP may not play a significant role in MTX response or safety in RA patients.

This study did not examine the association between the rs2231137 SNP and time to MTX discontinuation. However, using the Least Absolute Shrinkage and Selection Operator (LASSO) penalized regression, a previous study found an association between the rs2231137 SNP and time to discontinuation of MTX due to the appearance of ADRs and reported that this result may be a false negative in univariate [24]. Despite the potential importance of the present results, we acknowledge that our study has some limitations. First, our sample size was relatively small, primarily due to the limited availability of patients who met the strict inclusion criteria, even though the study was conducted as a multicenter investigation across multiple hospitals. This limitation may affect the generalizability of our findings to broader populations of RA patients. Second, reliance on retrospective data collection introduces the potential for underreporting, which could affect the completeness and reliability of the data. Finally, distinguishing between symptoms that are linked to MTX toxicity and those that are related to the patient’s overall health condition presents a challenge, making it difficult to correlate the rs2231137 SNP with MTX toxicity symptoms.

Overall, this article may provide valuable insights into the potential association between the 34G>A (rs2231137) polymorphism and clinical outcomes in RA patients treated with MTX. However, more research is needed to confirm our findings and to address the limitations of our study. In conclusion, to the best of our knowledge, this study is the first to examine the relationship between the 34G>A (rs2231137) SNP and MTX efficacy and/or toxicity in patients with RA using linear regression. The results indicate no link between the rs2231137 polymorphism in the *ABCG2* gene and MTX efficacy and/or toxicity in RA patients. Since research on the rs2231137 polymorphism in MTX pharmacogenetics for RA patients is limited, our findings may help guide future studies in this area. To confirm such results, further research with larger patient cohorts is necessary. Additionally, future studies could benefit from the inclusion of haplotype analyses.

## 4. Materials and Methods

### 4.1. Subjects and Study Design

This study, a retrospective, multicentric, case–control study, is a part of a large project designed to evaluate the influence of SNPs on MTX response in RA patients. The study focused on Saudi residents who had been diagnosed with RA based on the 2010 American College of Rheumatology/European League Against Rheumatism (ACR/EULAR 2010) criteria and were treated with methotrexate for at least 3 months. A total of 99 (78 women, 21 men) were collected from King Abdulaziz University Hospital in Jeddah and Armed Forces Hospital in Taif.

### 4.2. Ethical Approval and Ethical Consideration

The research protocol was approved by the Ethics Committee of the Biomedical Ethics Unit at the Faculty of Medicine, King Abdulaziz University, Jeddah (Reference No. 65-23; on 8 March 2023), and by the Research Ethics Committee of Armed Forces Hospitals, Taif (No. 2022-692; on 12 December 2022). All the participants were requested to sign written informed consent before collecting their data or samples and were free to withdraw at any time.

### 4.3. Data and Sample Collection

The study participants’ information was collected by using a predesigned electronic data sheet. The Sheet was structured into two sections; the first section includes demographic data, such as age, nationality, and gender, which were obtained orally. While the second section incorporated clinical data derived from their medical records, including the age of onset, MTX doses, duration of MTX use, use of SAIDs/NSAIDs, the results of RF, DAS-28, and ESR. Also, the common ADRs, if any, include Hair loss, GI, Complete blood count disturbance (CBC disturbance), and liver function disturbance (LFT disturbance).

Patients were classified into two groups, good responders and poor responders, based on their response to the MTX and/or appearance of any ADR. A good responder patient, receiving a stable MTX dose for at least 3 months with an ESR < 20 or CRP in the normal range, while a poor responder means the patient’s failure to reduce the ESR/CRP by at least 20% despite a minimum 6-month therapy with a dose of at least 15 mg/week [25]. This classification criterion was adopted to minimize variability across different centers, ensure consistency across our cohort, and eliminate ambiguity in patient classification. After the end of data collection, a venous blood sample (5 mL) was obtained from each participant in an EDTA tube and labeled with a unique code for privacy.

### 4.4. Genotype Analysis

#### 4.4.1. Gene and SNP Selection

This study focused on the *ABCG2* gene for the investigation. The BCRP 34G>A polymorphism (rs2231137) is located in exon 2, leading to a valine-to-methionine substitution (Val12Met) and the 421C>A polymorphism (rs2231142) is located in exon 5, resulting in the amino acid substitution from glutamine to lysine (Gln141Lys) were specifically chosen for this investigation based on their potential impact on the transport function of MTX and/or its associated toxicity, either by increasing or decreasing the gene activity [14,15,16].

#### 4.4.2. Target-Specific Primer Design

The exon sequence for the *ABCG2* gene was obtained from the Ensembl genome browser 109 (https://m.ensembl.org, accessed on 10 June 2025). The Primer3 software was utilized for designing the primers while considering their length, product size, GC content, and melting temperature (https://primer3.ut.ee, accessed on 9 September 2025). The forward and reverse primers for rs2231137 were (TGTGGCCCAGTTATTTCACT) and (CATTCCAAGTTGTGCCTGTC), respectively. The rs2231142 primers were (ACCACATTGCCTCACTTCAG) and (AGGCTTTGCAGACATCTATGG) forward and reverse, respectively.

#### 4.4.3. DNA Extraction and Purification

The genomic DNA of all participants was extracted from the whole blood samples (5 mL) using QIAamp DNA Blood Kits (Qiagen, Germantown, MD, USA; catalog # 51140) following the manufacturer’s instructions. The concentration of DNA (ng/mL), as well as the quality and quantity of DNA, were evaluated utilizing the NanoDrop™ 2000c Spectrophotometer (Thermo Scientific, Waltham, MA, USA). The procedure steps are described previously in Magadmi et al. [26].

#### 4.4.4. Polymerase Chain Reaction (PCR)

PCR amplification of all DNA samples was conducted prior to sequencing to confirm the presence and size of the product. The PCR protocol, including primer sequences and thermal cycling conditions, as well as the integrity of PCR products, was described previously in Magadmi et al. [26].

#### 4.4.5. Sanger Sequencing

The genetic analyzers 3500 (Applied Biosystems, Carlsbad, CA, USA) and (BigDye^®^ Terminator v3.1 Cycle Sequencing Kit (1000 rxns) 4337456, Thermo Scientific, Waltham, MA, USA) were used for Sanger sequencing according to the protocol recommended by the manufacturer. Magadmi et al. provide Further details of the sequencing procedure [27].

Electrophoresis workflow, sequencing standards, and performance-optimized polymer to separate the DNA and capillaries instruments were optimized by using the applied biosystem chemistry guide.

### 4.5. Statistical Analysis

The study population was divided into groups: First group: RA patients with poor response to MTX drug. Second group: RA patients with good response to MTX drug.

The patients’ demographic, laboratory, and clinical data were presented using descriptive statistics. Categorical variables were displayed as frequencies and percentages, while continuous variables were displayed as mean ± standard deviation (SD). To analyze differences between groups, Pearson’s chi-square test was used for categorical variables, and a *t*-test was used for continuous outcome variables. The Social Sciences Statistical Package (SPSS version 21) (IBM, Armonk, NY, USA) was used for statistical analysis.

SNPStats [28] (https://www.snpstats.net/) was used to conduct analyses of allele and genotype frequencies, an association between the SNP and response/clinical features, and the multiple inheritance models (dominant and recessive). The *p*-value of <0.05 was used to determine significance. The 95% confidence intervals (CI) and odds ratios (OR) were also calculated.

## Figures and Tables

**Table 1:** Clinical and demographic data of all participants.

**Character**		**Mean**	**SD**
Age (years)		52.7	12.9
Age of onset (years)		45	14
		**Frequency**	**Percentage**
Gender	Male	21	21.21
	Female	78	78.78
Nationality	Saudi	93	93.94
	Non-Saudi	6	6
**Clinical Feature**		**Frequency**	**Percentage**
Disease duration (years)	≥6 months	3	3
	6 m to 1 year	9	9
	>1 year	87	87.87
Duration of taking MTX	3 m to 1 year	16	16.2
	>1 year	83	83.8
Steroid or non-steroid	Yes	59	58.58
	No	42	41.41
Adverse drug reaction (ADR)	CBC disturbance	2	2
	LFT disturbance	3	3
	GI upset	7	7
	Hair loss	2	2
Patient classification based on MTX drug response	Good responders	44	44.44
	Poor responders	55	55.56
**Markers of Disease Activity**		**Mean**	**SD**
DAS-28 (N = 64)		3.1	1.1
ESR		27.5	17.5
**Markers of Disease Activity**		**Frequency**	**Percentage**
RF	+ve	69	69.70
	−ve	27	27.3
	Not known	3	3

SD, standard deviation; MTX, methotrexate; GI, gastrointestinal; CBC, Complete blood count; LFT, liver function test; RF, rheumatoid factor; DAS-28, Disease Activity Score-28; EER, erythrocyte sedimentation rate.

**Table 2:** Demographic data and Clinical parameters as predictors of MTX response in RA patients.

Factors			Good Responders N (%)	Poor Responders N (%)	*p*-Value
			44 (44.44)	55 (55.56)	
Age ^#^			51.90 (12.80)	53.38 (13.08)	0.576
Gender ^^^	Male		8 (38.1%)	13 (61.9%)	0.509
	Female		36 (46.2%)	42 (53.8%)	
Nationality ^^^	Saudi		44 (46.8%)	50 (53.2%)	0.040
	Non-Saudi		0 (0%)	5 (100%)	
Duration of MTX treatment ^^^	3 months to 1 years		10 (62.5%)	6 (37.5%)	0. 112
	>1 year		34 (41.0%)	49 (59.0%)	
Take Steroid or non-steroid ^^^	Yes		16 (27.6%)	42 (72.4%)	0.000
	No		28 (68.3%)	13 (31.7%)	
Adverse drug reaction (ADR) ^^^	CBC disturbance	Yes	1 (50.0%)	1 (50.0%)	0.873
		No	43 (44.3%)	54 (55.7%)	
	LFT disturbance	Yes	2 (66.7%)	1 (33.3%)	0.432
		No	42 (43.8%)	54 (56.3%)	
	GI upset	Yes	3 (42.9%)	4 (57.1%)	0.930
		No	41 (44.6%)	51 (55.4%)	
	Hair loss	Yes	2 (100%)	0 (0%)	0.110
		No	42 (43.3%)	55 (56.7%)	

N, patient numbers; GI, gastrointestinal; CBC, Complete blood count; LFT, liver function test. ^#^ Comparison was performed using an independent *t*-test. ^^^ Comparison was performed using chi-square.

**Table 3:** Genotype distributions and allele frequencies of ABCG2 polymorphisms.

Genotype/Allele	Frequency	Percentage
**rs2231137**		
CC	90	91
CT	7	7
TT	2	2
C	187	94
T	11	6
**rs2231142**		
GG	98	99
GT	1	1
G	197	99
T	1	1

**Table 4:** Genotype and allele frequencies of rs2231137 SNP among good responders and poor responders to MTX.

Genotype/Allele	Good Responder N (%)	Poor Responder N (%)	Adjusted OR (95% CI)	*p*-Value
	N = 44 (44.44)	N = 55 (55.56)		
CC	42 (95.5)	48 (87.3)	1	0.21
CT	1 (2.3)	6 (10.9)	5.49 (0.64)	
TT	1 (2.3)	1 (1.8)	0.91 (0.06)	
C	85 (97)	102 (93)	2.22(0.57)	0.25
T	3 (3)	8 (7)		

N, patient numbers; OR, odds ratio; CI, confidence interval. Data was analyzed using chi-square. OR estimated by logistic regression analysis after adjusting for regimen.

**Table 5:** Comparison of different rs2231137 SNP genotype models in MTX good responders and poor responders.

Model	Genotype	Good Responder N (%)	Poor Responder N (%)	Adjusted OR (95% CI)	*p*-Value
rs2231137					
Dominant	CC	42 (95.5)	48 (87.3)	1	0.15
	CT-TT	2 (4.5)	7 (12.7)		
Recessive	CC-CT	43 (97.7)	54 (98.2)	1	0.87
	TT	1 (2.3)	1 (1.8)		

N, patient numbers; OR, odds ratio; CI, confidence interval. Data was analyzed using chi-square. OR estimated by logistic regression analysis after adjusting for regimen.

**Table 6:** Associations between *ABCG2* SNP (rs2231137) and devolving ADRs.

ADR		rs2231137			*p*-Value
		CC	CT	TT	
Hair loss, N (%)	Yes	2 (100)	0	0	0.82
	No	88 (90.7)	7 (7.2)	2 (2.1)	
Adjusted OR (95% CI)		1	NA		
GI upset, N (%)	Yes	6 (85.7)	1 (14.3)	0	0.69
	No	84 (91.3)	6 (6.5)	2 (2.2)	
Adjusted OR (95% CI)		1	2.33 (0.24)	NA	
CBC disturbance, N (%)	Yes	2 (100)	0	0	0.82
	No	88 (90.7)	7 (7.2)	2 (2.1)	
Adjusted OR (95% CI)		1	NA		
LFT disturbance, N (%)	Yes	3 (100)	0	0	0.75
	No	87 (90.6)	7 (7.3)	2 (2.1)	
Adjusted OR (95% CI)		1	NA		

N, patient numbers; OR, odds ratio; CI, confidence interval; GI, gastrointestinal; CBC, Complete blood count; LFT, liver function test; NA: not applicable. Data was analyzed using chi-square. OR estimated by logistic regression analysis after adjusting for regimen.

**Table 7:** Associations between *ABCG2* SNP (rs2231137) genotypes with RF (N = 96).

Genotype/Allele	Positive RF, N (%)	Negative RF, N (%)	Adjusted OR (95% CI)	*p*-Value
	N = 69 (71.88)	N = 27 (28.12)		
rs2231137 (N = 96)				
CC	63 (91.3)	25 (92.6)	1	0.5
CT	4 (5.8)	2 (7.4)	1.26 (0.22)	
TT	2 (2.9)	0	NA	

N, patient numbers; OR, odds ratio; CI, confidence interval; RF, rheumatoid factor. Data was analyzed using chi-square. OR estimated by logistic regression analysis after adjusting for regimen.

**Table 8:** Associations of *ABCG2* SNP (rs2231137) genotypes with ESR level and MTX dose.

Characteristic	rs2231137			*p*-Value
	CC	CT	TT	
ESR level	27.52 (17.77)	22 (16.71)	33.5 (14.84)	0.643
MTX dose	15.16 (3.76)	13.57 (3.77)	15 (3.53)	0.559

Data was analyzed using a one-way ANOVA test. Data were presented as mean and standard deviation (SD). EER, erythrocyte sedimentation rate; MTX, methotrexate; SD, standard deviation.

**Table 9:** Association of *ABCG2* SNP (rs2231137) genotypes with the disease activity (N = 64).

Genotype	DAS-28	*p*-Value
CC	3.07(1.08)	0.292
CT	3.88(0.952)	
TT	2.43(0.1)	

Data was analyzed using a one-way ANOVA test. Data were presented as mean and standard deviation (SD). SD, standard deviation; DAS-28, Disease Activity Score-28.

## Data Availability

All relevant data are contained in the article, the original contributions presented in the study are included in the article, and further inquiries can be directed to the corresponding author.
